# Predictive Modeling of Phase Behavior of Reservoir Fluids under Miscible Gas Injection Using the Peng–Robinson Equation of State and the Aromatic Ring Index

**DOI:** 10.1021/acsomega.2c06813

**Published:** 2023-01-09

**Authors:** Ali A. AlHammadi, Mohammed I. L. Abutaqiya

**Affiliations:** † Chemical Engineering Department, Khalifa University of Science and Technology, P.O. Box 127788, Abu Dhabi127788, United Arab Emirates; ‡ Center for Catalysis and Separations, Khalifa University of Science and Technology, P.O. Box 127788, Abu Dhabi127788, United Arab Emirates; § ExxonMobil Technology and Engineering Company, Spring, Texas77389, United States

## Abstract

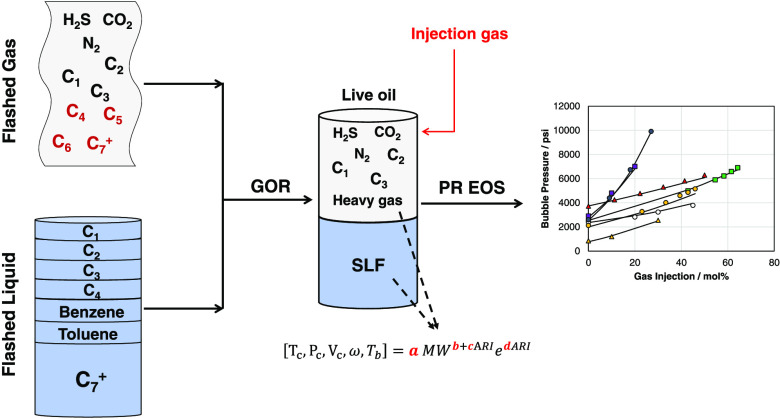

Improved correlations among critical temperature, critical pressure, and acentric factor are developed for an extensive database of hydrocarbons. The correlations rely on measurements of molecular weight and refractive index at ambient conditions and utilize the concept of the aromatic ring index (ARI) recently developed by Abutaqiya et al. as a distinctive characterization factor for nonpolar hydrocarbons [AbutaqiyaM. I. L.Aromatic Ring Index (ARI): A Characterization Factor for Nonpolar Hydrocarbons from Molecular Weight and Refractive Index. Energy Fuels2021, 35( (2), ), 1113−1119.]. The new correlations are then implemented for modeling the phase behavior of a variety of oils under miscible gas injection in a fully predictive manner using the Peng–Robinson equation of state (PR EOS). The results indicate that the proposed modeling framework yield accurate predictions for bubble pressure of oil/gas blends with an average absolute deviation of 6.4% for a wide variety of oils and injection gases including lean, rich, H_2_S, CO_2_, and N_2_. Additionally, an interesting crossover behavior in the phase envelope of live oils under CO_2_ injection is observed using PR EOS. This behavior has been previously reported in the literature for modeling results using PC-SAFT EOS and seems to be characteristic of CO_2_.

## Introduction

Equations of state are convenient and widely used thermodynamic models for predicting phase behavior and physical properties of fluids. The development and improvement of these equations are of utmost interest in various industries. The popularity of equations of state in these industries is motivated by their applicability over a wide range of temperatures and pressure and also by their simplicity. One of the earliest equations of state was the one developed by van der Waals,^1^ who used it for gases and liquids. Van der Waals also proposed attractive and repulsive terms. Fundamental analysis of those terms in the van der Waals (VdW) equation of state and its deep theoretical roots shows the secrets of its popularity and the difficulty in replacing them. Since then, many researchers have tweaked these terms for better predictions that ended up with a large number of equations of state. Kontogeorgis et al.^2^ presented a thorough analysis of the practicality of van der Waals equation of state for pure and mixtures while an excellent review of the cubic equations of state (based on VdW) is presented by Wei and Sadus.^3^

The two most popular and widely used ones are the Peng–Robinson (PR)^4^ and the Soave–Redlich–Kwong (SRK)^5^ equations of state. Their simplicity, quick implementation, and relative accuracy made them widely popular, especially in the oil industry. Both equations follow the same standard formula and require three parameters for every component, which are critical temperature (*T*_c_), critical pressure (*P*_c_), and acentric factor (ω). Therefore, accurate critical properties are necessary for an accurate prediction of thermodynamic and physical properties as well as for determining the optimum design and control in all equipment.

Critical properties play an important role in every step of process design and simulation. Examples of these include phase behavior modeling, flash calculations,^6^ modeling fluid transport in oil and gas production,^7^ estimation of saturation, and thermal and transport properties.^8^

Although these parameters can be obtained experimentally, so doing so may not always be feasible due to different factors. These include the high cost of experiments, decomposition of heavy components, and impurities within the sample. Therefore, alternative models were developed to estimate these thermophysical properties. In 1933, Watson and Nelson were the first to develop empirical models and charts to approximate the properties of both pure compounds and the heavy petroleum fraction.^9^ Many have followed suit to develop more accurate models or specific cases. These models can be divided into two categories. The first one is group contribution (GC) theory, where properties are calculated based on the molecular structure, atom relative positions, and steric interactions, thus providing a robust and general approach. These models range from Riedel^10^ and Lydersen^11^ to Poling et al.,^12^ Gharagheizi et al.^7^ and Avaulee et al.^13^ The disadvantage of this method is that substantial structural information is needed, which is especially challenging when dealing with crude oils.

The second and most common approach is to depend on easily measured bulk properties such as molecular weight, specific gravity, refractive index, and boiling temperature to predict physiochemical properties. This method provides a significant advantage including the ability to predict these properties based on limited data. However, this comes at the cost of accuracy. A summary of some of the common and recent models is presented in Table 1.

**Table 1:** Summary of Commonly Used Models to Predict Critical Properties for Pure and Pseudocomponents

model	input	output	comments
Riazi and Daubert^14^	specific gravity and normal boiling point (or molecular weight)	critical properties	recommended only for hydrocarbons (CN# 1–20) with MW of 70–300 and normal Tb of 299–616 K
Lee and Kesler^15^	specific gravity and boiling point	critical temperature and pressure	*M*_W_ of 70–700. Data above C18 were not based on experimental evidence
Cavvet^16^	boiling point and API gravity	critical pressure and temperature	no mention of the source or type of data used
Winn^17^ and Mobil^18^	boiling point (or Watson characterization factor) and the specific gravity (or API gravity)	physical prop. for pure and pseudocritical petroleum fraction	Riazi^19^ converted these nomographs to equations that were later reported by Sim and Daubert^18^
Tsonopoulos^20^	specific gravity and boiling point.	critical pressure and temperature	recommended for coal liquids and aromatic-rich fractions
Hall and Yarborough^21^	molecular weight and specific gravity	critical volume	specific to critical volume
Hosseinifar and Jamshidi^22^	specific gravity and molecular weight (or boiling point)	critical properties	a variation exists for petroleum applications where the parameters were correlated to molecular weight and density instead
Evanglista and Vargas^23^	molecular weight and factor of refractive Index	critical properties and acentric factor	refractive index is used instead of the density

**Figure 1:**
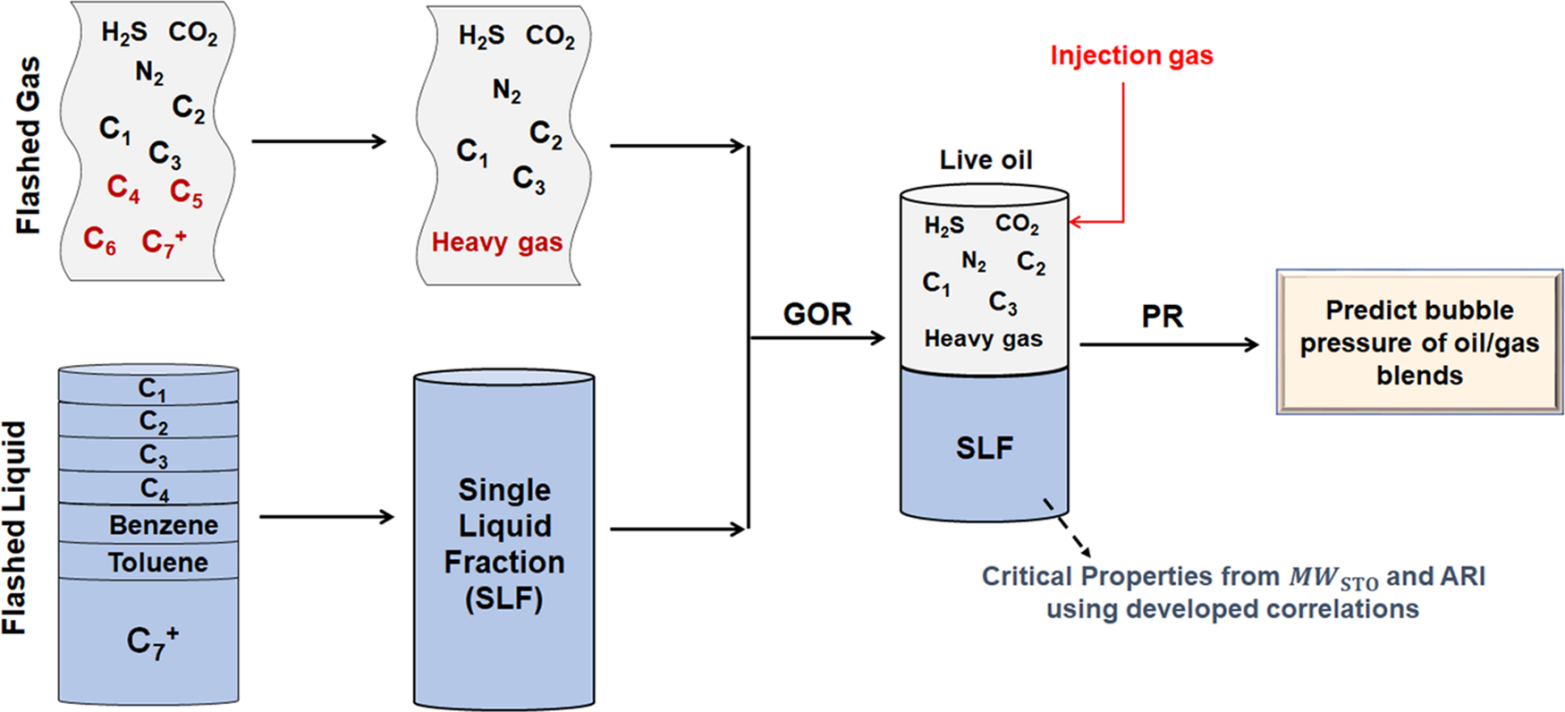
Schematic of the methodology to predict bubble pressure for various crude oils under gas injections. The flashed gas is characterized by lumping the C4+ into heavy gas, while the flashed liquid is all combined into a single liquid fraction (SLF). They are merged together based on the gas–oil ratio (GOR). The critical properties are estimated from correlations and used in PR EoS to predict bubble pressure. A detailed characterization is provided in the Supporting Information.

One main disadvantage of most previous models is that they are often accurate only for certain homologous families. In this work, we present a combination of two approaches. Empirical correlations are developed for normal boiling point, critical temperature, critical pressure, and acentric factor as a function of molecular weight and aromatic ring index (ARI). The aromatic ring index (ARI) can provide an indication of the number of aromatic rings present in the molecular structure and is shown to clearly distinguish between different families of hydrocarbons, including n-alkanes, cycloalkanes, benzene derivatives, and naphthalene derivatives.^24^ Moreover, these correlations were used to complement the semipredictive group contribution concept so that it can be also applied in a fully predictive manner. Predictions of various crude oils are presented as well according to the schematic presented in Figure 1. A step-by-step characterization is presented in the Supporting Information.

## Model Development

Wang et al.^25^ expressed critical properties and acentric factor as a function of molecular weight and refractive index at standard conditions. Evangelista et al.^23^ made a modification to represent them using *F*_RI_, which is a function of the refractive index. Physiochemical properties follow consistent change behavior within the same homogeneous family. Therefore, a factor that can distinguish between different hydrocarbon families can provide improved prediction accuracy such as the previously introduced aromatic ring index (ARI). As described by Abutaqiya et al.,^24^ the ARI is designed such that it yields a value of 0 for n-alkanes and 2 for naphthalene derivatives. With this definition, it is found that benzene derivatives yield a value of ARI = 0.95, which is roughly an indication of the number of aromatic rings in this class of components. Based on its successful classifications and the fact that many physicochemical properties of hydrocarbons are family-oriented, the ARI can be used to infer which group of hydrocarbons it is based on. On the other hand, the molecular weight provides an indication of its size contribution. The proposed model is
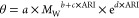
where *M*_W_ is the molecular weight, ARI is the aromatic ring index, and θ is the property to be predicted. The values of *a*, *b*, *c*, and *d* are empirical coefficients summarized in Table 2.

**Table 2:** Parameters for the Proposed Method to Determine Normal Boiling Point, Critical Properties, and Acentric Factor and the Absolute Average Percent Error (AAPE)

properties	*a*	*b*	*c*	*d*	average absolute error (%)
*T*_b_ (K)	3.520	0.517	–0.119	0.684	1.9
*T*_c_ (K)	4.583	0.368	–0.133	0.798	2.1
*P*_c_ (bar)	7.085	–0.820	–0.216	1.376	3.6
*V*_c_(mL/mol)	1.435	1.001	0.174	–1.007	3.6
ω	–5.642	0.979	–0.030	0.127	6.5

## Model Evaluation

The model used is relatively simpler than other available models in literature while providing similar or better predictions. This is especially important for heavy oil characterization. A comparison of the error produced is presented in Table 3. Other commonly used correlations are Soreide for the boiling point,^26^ Lee–Kesler for critical temperature, critical pressure and acentric factor,^15^ and Hall–Yarborough for critical volume.^21^ Another recent correlation by Evangelista and Vargas et al. is also compared.^23^

**Table 3:** Comparison between the Proposed Method vs Currently Used Equations^a^

		common method		
property	this work (%)	method name	error (%)	Evangelista (%)
boiling point	1.9	Soreide	4.1	
critical temperature	2.1	Lee–Kesler	1.4	2.4
critical pressure	3.6	Lee–Kesler	6.2	5.4
critical volume	3.6	Hall–Yarborough	3.6	5.5
acentric factor	6.5	Lee–Kesler	8.9	7.6

a The proposed correlation within this paper shows consistent improvement over the commonly used correlations. The error is reported as the average absolute percent error (AAPE).

**Figure 2:**
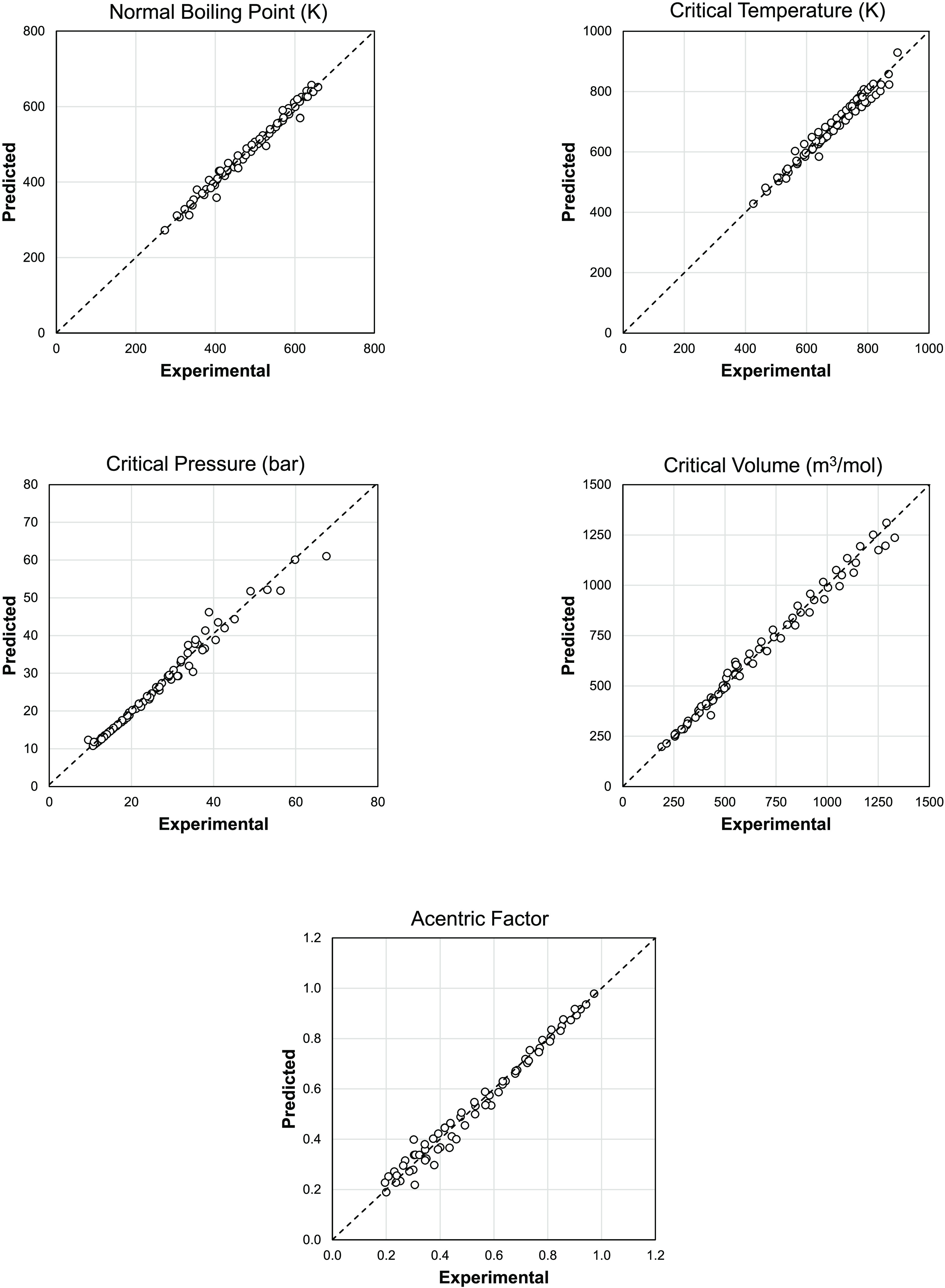
Comparison between the experimental and predicted values for the different physiochemical properties using the developed correlations. The predicted values are in good agreement with the experimental values, indicating accurate prediction for the various hydrocarbons studied. The AAPE for the different physiochemical properties is reported in Table 2.

Clearly from Table 3, the proposed correlation is on par and better compared to other alternative correlations that are currently being used in the literature. Parity plots for the different properties are shown in Figure 2.

From Table 3 and Figure 2, we can clearly see that the new correlations are accurate and offer better predictions for various hydrocarbons. It is also worth mentioning that the critical properties and acentric factor were tested on other components and yielded similar results in the ARI range of 0–3.5. To further test the capability of the proposed models, the above models will be used to calculate the critical properties and acentric factor for pseudocomponents and then the Peng–Robinson equation of state will be used to predict the bubble pressure of the blends while conserving their density predictions.

## Crude Oil Database

The predictive capability of the single liquid fraction (SLF) modeling approach applied in this work is tested using a database consisting of 35 live oils collected from the literature. The PVT properties of the 35 live oils are given in the Supporting Information. These oils come from various oil reservoirs around the world. The reader is referred to the respective references for more details on the fluids.^27^

## Results and Discussion

The flashed gas is characterized as light gases including N_2_, CO_2_, H_2_S, C_1_, C_2_, and C_3_ while C_4+_ is lumped together as heavy gas. On the liquid side, the flashed liquid is combined as a single component referred to as the single liquid fraction (SLF). Using the Mw and ARI of the live oil of the database, the critical parameters can be calculated using the developed correlations. A detailed step-by-step characterization of Crude Oil B59 is presented in the Supporting Information. A number of PVT experiments are usually conducted to understand the volumetric changes in the live oil as a function of pressure, temperature, and composition (i.e., gas injection). In this section, the SLF modeling approach is applied to study the effect of these operating variables on the bubble pressure of the 35 live oils in the database (the reader is referred to Abutaqiya et al.^27^ for details about these crudes). Additionally, full vapor–liquid phase envelopes predicted by the model are analyzed and compared to the available experimental data. The compositions and simulation parameters used for the 35 characterized live oils can be found in the Supporting Information.

**Figure 3:**
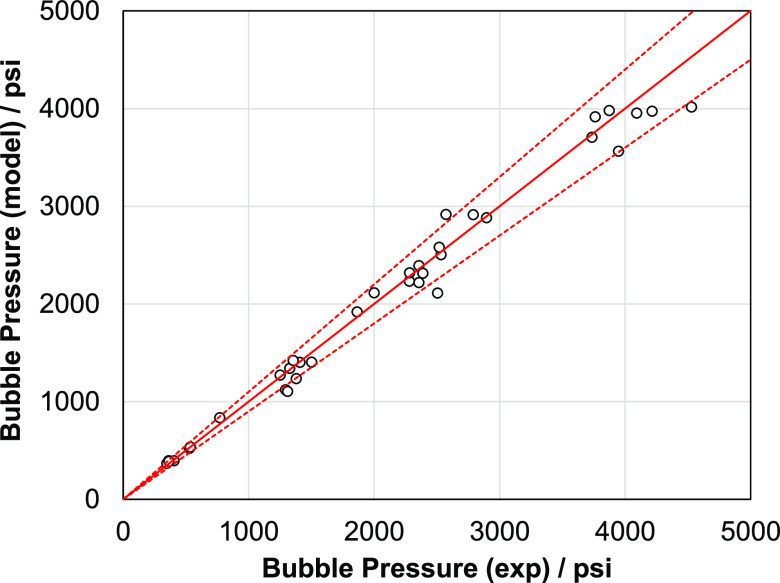
Predicted bubble pressure using the SLF approach for live oils at reservoir temperature presented as a parity plot [AAPD: 6.4%]. Dashed lines represent the ±10% error range. The solid line on the panel represents the diagonal line, which corresponds to a perfect model prediction.

Figure 3 shows a parity plot for the predictions of live oil bubble pressure at reservoir temperature for the 35 fluids studied in this work. Lines for the ±10% deviation are also shown for demonstration. With the exception of one case, the bubble pressure of live oils is predicted with an AAPD of less than 10% across the wide range of bubble pressures studied: 24.3–274.5 bar (352–3980 psi).

**Figure 4:**
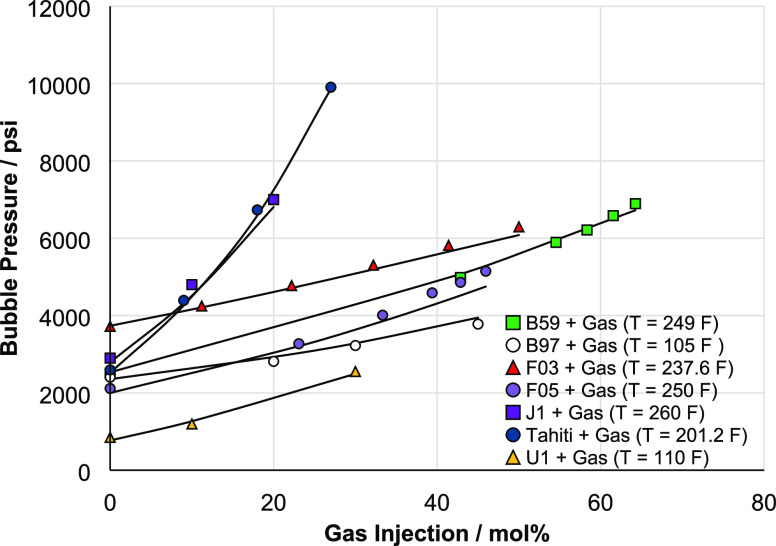
Predicted bubble pressure using the SLF approach for live oil with various injection gases [AAPD: 3.6%]. The model shows excellent prediction capabilities for different hydrocarbons with gas injections. These results are part of swelling test experiments.

Figure 4 shows the predictions of bubble pressure as a function of gas injection for various crude oil + injection gas blends. These results are parts of swelling test experiments where gas is injected into the crude oil, causing it to swell (volume expansion). This will substantially increase its saturation pressure as more gas is dissolved in it.^28^ The SLF model shows excellent predictive capabilities for a wide range of injection gases including N_2_, CO_2_, and hydrocarbon mixtures.

To further test the predictive capability of the SLF model, the case of crude S14 in which there exists a known experimental inaccuracy in one of the measured bubble pressures is investigated. In this case, two similar bubble pressures (2029.2 and 2054.2 psi, respectively) were reported by the service laboratory for blends with 10 and 20% hydrocarbon gas injections. This case was further studied by Vargas et al.^29^ using a SARA-based characterization methodology. The authors indicated that the experiment with 10% gas injection is flawed; a result that is further corroborated by our analysis.

**Figure 5:**
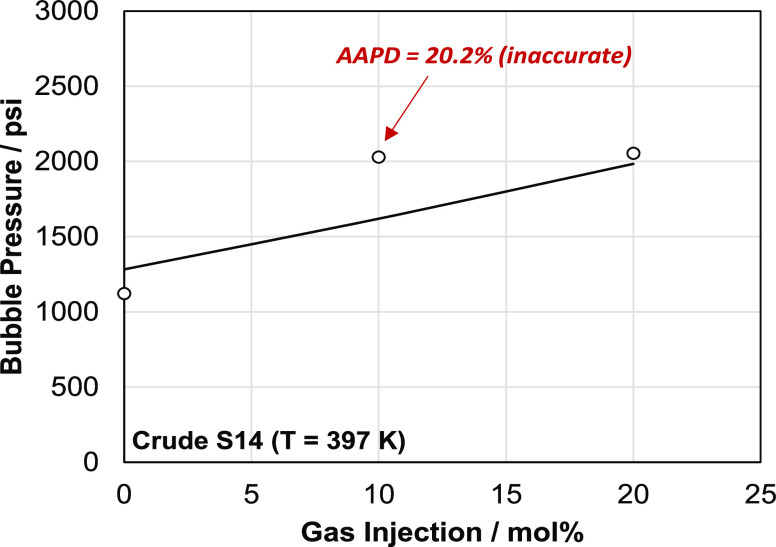
Predicted bubble pressure as a function of gas injection for S14 at 397 K using SLF. The reported bubble pressure at 10% injection is inaccurate. The SLF model developed in this work can detect this inaccuracy. APD: absolute percent deviation. Gas composition (mol %): N_2_: 0.65, H_2_S: 0.031, CO_2_: 6.73, C1: 60.57, C2: 13.70, C3: 10.19, and C4+: 8.13.

Figure 5 shows the bubble pressure prediction for crude S14 as a function of injection gas using the predictive SLF model developed in this work. The figure indicates that there is a substantial deviation in the bubble pressure prediction at 10% injection, which is in agreement with the results of Vargas et al.^29^ An important differentiator is that our results shown here in Figure 5 are completely predictive. In contrast, Vargas et al.^29^ used the bubble pressure and saturation density of the live oil to parameterize their model. In addition, Stock tank oil is presented in our approach as a single pseudocomponent fraction (SLF). This ensures that no detailed characterization is needed for the liquid fraction as is the case of a SARA approach where they are described as three distinct pseudocomponents (saturates, aromatics + resins, and asphaltenes).

## Modeling the VLE Phase Envelope

**Figure 6:**
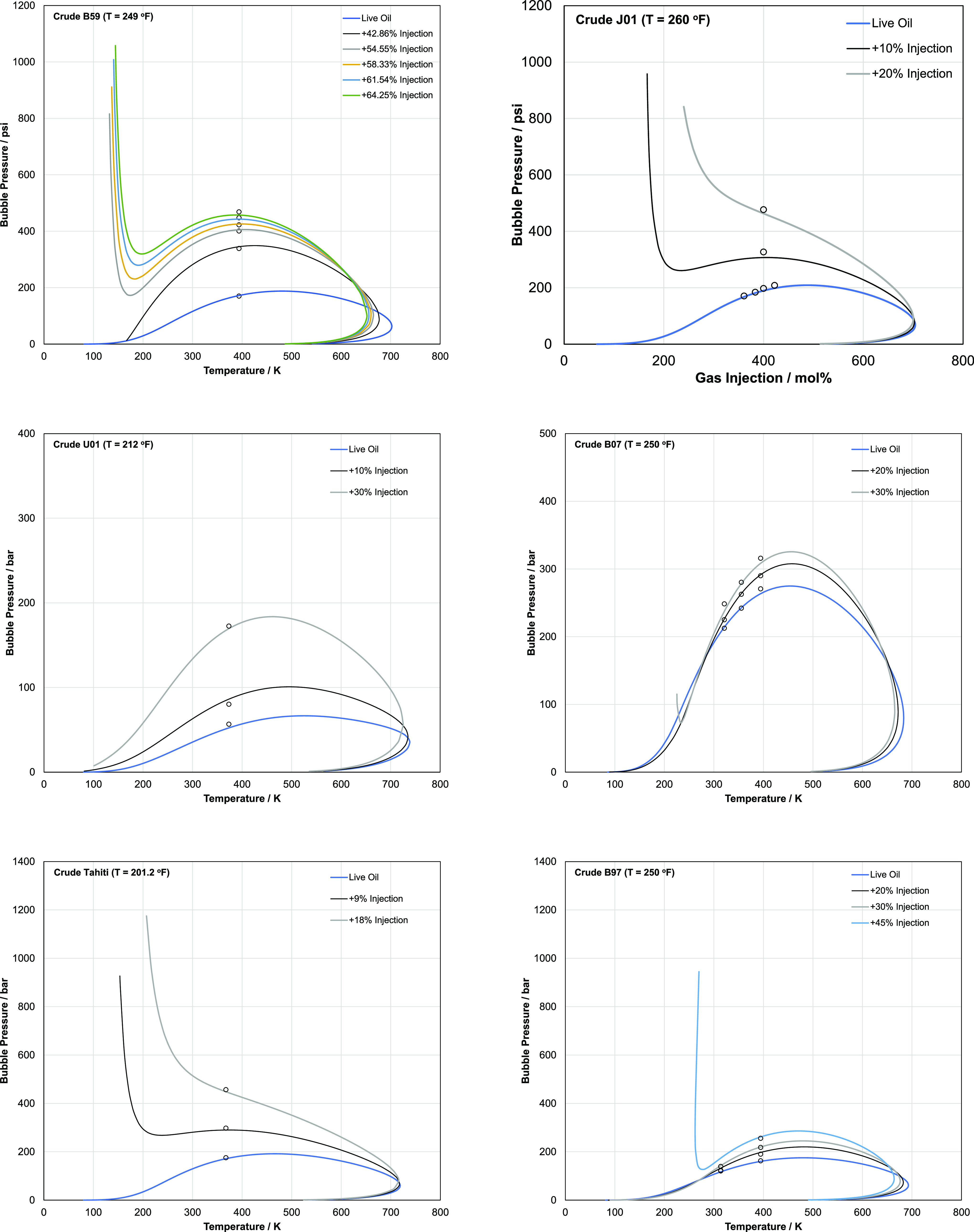
Predicted P–T phase envelopes for vapor–liquid equilibria using PR with the SLF lumped solvent approach for various live oils and their gas blends. Lines represent the PR predictions. Filled circles represent the experimental data. Open squares represent the predicted critical point. The SLF model can capture with reasonable accuracy the temperature dependence of the bubble pressure for reservoir fluids under various gas injections.

To gain more insight into the capability of the SLF model in representing the phase behavior of reservoir fluids, the full P–T phase envelopes for vapor–liquid equilibria are investigated in this section. Figure 6 shows the predicted P–T phase envelopes for various reservoir fluids and their gas blends using the SLF approach. As mentioned before, the dew point curves shown in Figure 6 are not expected to accurately represent the true dew curve because the dew point is driven by the components in the heaviest fraction of the crude oil and the SLF approach lumps all of these fractions into a single pseudocomponent. As shown in Figure 6, the model can generally capture with reasonable accuracy the temperature dependence of the bubble pressure for reservoir fluids.

Interestingly, it is observed from Figure 6f that the PR is able to capture the CO_2_ injection behavior and provides a reasonable trend match and does not provide crossover behavior as predicted by PC-SAFT. A detailed analysis of CO_2_ crossover behavior was carried out by Vargas et al.^30^ and Arya et al.^31^

## Error Analysis

The resulting PR simulation parameters for all dead oils, petroleum fuels, and live oils are given in the Supporting Information. The following statistical measures are used to analyze the errors in the predictions of the SLF-ARI model
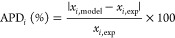

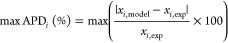

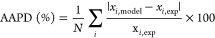

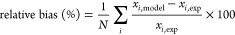

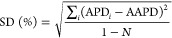
where *x*_*i*,Model_ is the predicted value, *x*_*i*,Exp_ is the experimental value, *N* is the number of data points, APD*_i_* is the absolute percent deviation in the prediction of data point *i*, AAPD is the average APD, and SD is the standard deviation of the sample of absolute errors. The statistical error analysis of the model predictions for all fluids investigated is shown in Table 4.

**Table 4:** Error Analysis for the Prediction of Bubble Pressure of Oil/Gas Blends Using the Proposed Approach

	*N* [−]	AAPD [%]	rel. bias [%]	max APD [%]	SD [%]
live oils	35	4.9	0.7	18.7	7.4
oil/gas blends	51	7.0	–2.8	21.1	6.2
overall	86	6.4	–1.1	21.1	5.8

The statistical analysis shows an overall AAPD of 6.4% for bubble pressure for all 35 investigated fluids in a total of 86 blends. Note that the live oil is a result of various combinations coming from heavy gas, single pseudocomponent representing liquid fraction, measured GOR, molecular weight, and density of STO. Therefore, it is expected that the propagated error from combining these measurements and calculations will impact the overall accuracy of the model. experimental error from all these measurements is expected to affect the predictive capability of the model. Considering the predictive capabilities of the proposed SLF with limited information, the obtained error is very reasonable and demonstrates a strong predictive capability of the model.

The maximum deviations in bubble pressure of live oils (18.7%) and oil/gas blends (21.1%). Note that among the 35 studied crudes, only one crude (S1 crude) shows more than a 10% deviation in the predicted bubble pressure of the live oil from the experimental value as can be seen in Figure 2. Based on the overall AAPD of 6.4%, it can be concluded that the SLF model is generally capable of capturing the bubble pressure for both live oil and under various types of gas injections.

It is also worth noting that in all of the previous 35 crude oils, the binary interaction coefficients (*k*_ij_) were fixed (available in the Supporting Information). These were based on the reported values by Abutaqiya et al.^27^ and further fitted to one crude oil (B59) density and phase behavior. Further tuning of these binary interaction coefficients can enhance the accuracy of the model. The most sensitive *k*_ij_ is the value between the SLF pseudocomponent and methane (C1), which is due to the amount of both components. A sensitivity analysis has shown that all crude oils and their mixtures blends can be obtained with less than 3% error upon the tuning of this *k*_SLF-C1_ for a range of −0.05–0.07. This, however, will lessen the predictive capabilities of the proposed model, which is the reason that the binary interaction coefficients were assumed to be consistent for all crude oils.

## Conclusions

One of the challenges facing thermodynamic modeling using cubic equations of state is the inability to always obtain necessary parameters experimentally. Fortunately, the approaches of group contribution theory and empirical models provide a tool to obtain these parameters. However, these approaches are limited especially when it comes to pseudocomponents or heavy crude oil and the need to know the original homogeneous family. Therefore, this work presents new correlations that are more systematic to characterize and predict these needed properties for both pure and pseudocomponents. This approach is based on the aromatic ring index (ARI) and molecular weight. The ARI will provide information about the molecular structure and family that the pseudocomponent belongs to while the molecular weight provides information about its size.

The SLF approach for characterization^25^ combined with the developed correlations for parameterizing the heavy fraction (SLF-ARI) produces a fully predictive thermodynamic modeling approach that does not require SARA analysis, H/C ratio, or tuning to experimental data. The SLF modeling approach yielded AAPD values in the 35 live oils bubble pressure of 4.90%. Additionally, the SLF modeling framework was shown to be capable of detecting experimental discrepancies in live oil bubble pressure measurements.

In addition, this work reinforces the concept that the aromatic ring index is a great indicator of the behavior of the components. Combining it with molecular weight showed the capability to accurately estimate critical properties and acentric factor for a wide variety of components.

Such an approach can be further expanded to other characterization techniques and not necessarily limited to cubic equations of state as the PR. One example of this is asphaltene, where ARI was shown to be able to replace both tuning parameters of molecular weight and aromaticity. Through this work, we aim to set a foundation for a characterization technique that is more systematic and can be implemented for a variety of complex systems. Such information would be of great value in the petrochemical industry.

## Supporting Information Available

Single liquid fraction (SLF) characterization method and parameterization using the aromatic ring index (ARI); simulation parameters for all investigated petroleum fuels and crude oils; and composition and binary interaction parameters used for modeling live oils (PDF)
