# Supplementary material for: Predictive Modeling of Phase Behavior of Reservoir Fluids under Miscible Gas Injection Using the Peng–Robinson Equation of State and the Aromatic Ring Index
Source: ACS Omega. 2023 Jan 9;8(3):3270–7. doi: 10.1021/acsomega.2c06813 (PMC9878645; doi:10.1021/acsomega.2c06813)
Supplement: Supplementary file 1 — ao2c06813_si_001.pdf [file ao2c06813_si_001.pdf]

Supplementary Information

Predictive Modeling of Phase Behavior of Reservoir Fluids Under Miscible Gas Injection Using Peng-Robinson Equation of State and Aromatic Ring Index

Ali A. AlHammadi[1,2] and Mohammed I. L. Abutaqiya[3]*

[1] Chemical Engineering Department, Khalifa University of Science and Technology, P.O. Box 127788, Abu Dhabi, United Arab Emirates,

[2] Center for Catalysis and Separations, Khalifa University of Science and Technology, P.O. Box 127788, Abu Dhabi, United Arab Emirates

[3] ExxonMobil Technology and Engineering Company, Spring, TX, USA.

In this supplementary document, a step-by-step example for the crude oil characterization is presented using the single liquid fraction (SLF) method and Aromatic Ring Index (ARI) parameterization method.

Also, the composition of the crude oils and injected gases are presented. Furthermore, numerical values for the experimental and simulation data using the SLF method are tabulated for all 10 crudes studied. Finally, the simulation parameters using the SARA-based method are presented.

Example for the SLF characterization:

In this section the characterization procedure for Crude B59 is demonstrated. The properties and experimental values required for the characterization are shown in Table S1.

Table S1. Input parameters for the characterization of Crude B59

**Property**	**Crude B59**
**STO MW (g/mol)**	208
**STO API gravity**	38
**STO density (kg/m^3)**	834.7
**GOR (Sm^3/m^3)**	164
**Reservoir Psat (Mpa)**	17.35
**Reservoir Tsat (^{o}C)**	121.1
**Saturation density (kg/m^3)**	647.6

Step 1: characterize gas phase by lumping all C4+ fractions into a single pseudo-fraction (heavy gas) as shown in Table S2.

Table S2. Composition of the bottomhole flashed gas for Crude B59 and the composition of the characterizedgas phase

Flashed gas				Characterized gas phase		
**Component**	**MW**	**z_i (mol%)**		**Component**	**MW**	**z_i (mol%)**
**N_2**	28.01	0.19		N_2	28.01	0.19
**CO_2**	44.01	4.09		CO_2	44.01	4.09
**H_2S**	34.08	0		H_2S	34.08	0
**C_1**	16.04	53.94		C_1	16.04	53.94
**C_2**	30.07	10.61		C_2	30.07	10.61
**C_3**	44.1	9.99		C_3	44.1	9.99
**iC_4**	58.12	2.36		Heavy gas	72.3	21.19
**nC_4**	58.12	6.05				
**iC_5**	72.15	2.67				
**nC_5**	72.15	3.36				
**C_6**	86.18	4.26				
**C7+**	101	2.48				

Step 2: Combine the gas phase and liquid phase fractions using the zero flash GOR to obtain the live oil composition. In order to combine the two phases, we convert the GOR from Sm^3/m^3 to mol gas/mol liquid using the experimental properties reported as follows:

$$moles\ of\ gas\ per\ m^3 = GOR\left(\frac{Sm^3}{m^3}\right) \times 42.302\frac{mol}{Sm^3} = 6937.66\frac{mol}{m^3}$$

$$moles\ of\ liquid\ per\ m^3 = \frac{\rho_{STO}\left(\frac{kg}{m^3}\right)}{MW_{STO}\left(\frac{kg}{kmol}\right)} \times 1000 = 4012.9\frac{mol}{m^3}$$

$$\frac{mol\ gas}{mol\ liquid} = \frac{6937.6}{4012.9} = 1.728$$

Therefore, in 100 mol live oil there is 63.31 mols of gas (composed of the components in Table S2) and 36.69 mols of liquid (composed of a single liquid fraction). The final live oil composition is shown in Table S3.

Table S3. Final composition and molecular weight of the characterized live oil

Component	MW	z_i (mol%)
N_2	28.01	0.12
CO_2	44.01	2.59
H_2S	34.08	0
C_1	16.04	34.15
C_2	30.07	6.72
C_3	44.1	6.33
**Heavy gas**	72.3	13.41
**SLF**	208	36.68

Step 3: Obtain the critical properties for all components and pseudo fractions. For pure components, the critical pressure, temperature, volume, and acentric factor are available and reported in Table S4.

Table S4. Critical properties for pure pre-defined components.

Name	MW (g/mol)	Tc (K)	Pc (Bar)	Vc (cm^3/mol)	Acentric factor (-)
N_2	28.01	126.20	34.00	89.21	0.038
CO_2	44.01	304.21	73.83	94.00	0.224
H_2S	34.08	373.53	89.63	98.50	0.094
**C1**	16.04	190.56	45.99	98.60	0.012
**C2**	30.07	305.32	48.72	145.50	0.099
**C3**	44.10	369.83	42.48	200.00	0.152

The heavy gas pseudo fraction is assumed to be composed of pure alkanes and will therefore have an ARI of around 0. To confirm this, the ARI is calculated for both Heavy gas and SLF using its molecular weight and density at 20 ^{o}C (or Refractive Index Factor) from the correlation below:

$$ARI = f(\boldsymbol{MW},\boldsymbol{F_{RI}}) = \frac{2\left[\frac{\boldsymbol{MW}}{\boldsymbol{F_{RI}}} - (3.5149\boldsymbol{MW} + 73.1858)\right]}{(3.5074\boldsymbol{MW} - 91.972) - (3.5149\boldsymbol{MW} + 73.1858)}$$

The FRI can be calculated typically from the refractive index. However, if it is not available, the Lorentz-Lorenz expansion proposed by Vargas and Chapman is used to calculate $F_{RI,20}$ from the reported density:

$$F_{RI,20} = 0.5054\rho_{20} - 0.3951\rho_{20}^{2} + 0.2314\rho_{20}^{3}$$

Using the density at 20 °C for Heavy gas (0.6329 g/cm3) and SLF (0.8314 g/cm3), the FRI can be calculated. Using them along with the molecular weights reported above, the ARI can be obtained as shown in Table S5.

Table S5. ARI calculated based on FRI and MW of the heavy gas and SLF pseudo-components.

Component	Density at 20 °C (g/cm³)	FRI_{20}	MW	ARI
**Heavy Gas**	**0.6328**	**0.2202**	**72.33**	-0.012
**SLF**	**0.8314**	**0.2801**	**208.00**	0.739

The different critical properties and acentric factor can be calculated from the expressions below (copied here for convenience):

$$\theta = a * MW^{b + c * ARI} * e^{d * ARI}$$

where MW is the molecular weight, ARI is the Aromatic Ring Index and θ is the property to be predicted. The values of a, b, c, and d are empirical coefficients summarized in Table S6 (**Error! Reference source not found.** in the original paper).

Table S6. Parameters for the proposed method to determine normal boiling point, critical properties, and acentric factor and the absolute average percent error (AAPE)

Properties	A	b	c	d	AAPE (%)
$T_b(K)$	3.520	0.517	-0.119	0.684	1.9
$T_c(K)$	4.583	0.368	-0.133	0.798	2.1
$P_c(bar)$	7.085	-0.820	-0.216	1.376	3.6
$V_c(ml/mol)$	1.435	1.001	0.174	-1.007	3.6
ω	-5.642	0.979	-0.030	0.127	6.5

Based on the values above, the calculations yield the following critical properties and acentric factor as shown in Table S7.

Table S7. Critical parameters based on the ARI and MW for the pseudo components for Crude oil B59.

Component	$MW(g/mol)$	$T_b(K)$	$T_c(K)$	$P_c(bar)$	$V_c(ml/mol)$	ω
**Heavy Gas**	72.3	308.64	471.21	35.51	305.84	0.2347
**SLF**	208	554.46	742.50	17.73	829.12	0.6448

The final composition and critical parameters for the live oil (Crude B59) are shown in Table S8.

Table S8. Final composition and simulation parameters for the characterized Crude Oil B59.

Component	$MW(g/mol)$	z_i (mol%)	$T_c(K)$	$P_c(bar)$	$V_c(ml/mol)$	ω
N_2	28.01	0.12	126.2	34	89.21	0.0377
CO_2	44.01	2.59	304.21	73.83	94	0.2236
H_2S	34.08	0.00	373.53	89.63	98.5	0.0942
C_1	16.04	34.15	190.56	45.99	98.6	0.0115
C_2	30.07	6.72	305.32	48.72	145.5	0.0995
C_3	44.1	6.33	369.83	42.48	200	0.1523
**Heavy Gas**	72.3	13.41	471.21	35.51	305.84	0.2347
**SLF**	208	36.68	742.50	17.73	829.12	0.6448

This was done for all 35 studied crudes. The composition of characterized crude oils are shown in Table S9.

Table S9. Characterized composition of the 35 studied crude oils.

	N_2	CO_2	H_2S	C1	C2	C3	C4+	SLF
**B16**	0.12	2.65	0.00	37.95	6.81	5.90	13.29	33.28
**F07**	0.00	2.13	0.00	31.28	7.51	6.93	11.00	41.15
**F08**	0.08	1.82	0.00	32.17	7.63	7.22	11.53	39.56
**F09**	0.25	3.60	2.32	47.64	6.50	4.50	7.33	27.86
**F10**	0.40	2.55	0.36	47.24	6.94	4.81	7.66	30.05
**M1**	0.32	0.70	3.55	32.60	8.42	6.73	7.17	40.51
**R2W1**	0.21	0.75	0.51	6.05	2.59	5.83	19.25	64.81
**R2W2**	0.88	1.34	0.00	5.63	2.51	4.60	18.01	67.03
**R2W3**	0.30	0.01	0.00	7.14	1.54	3.71	20.15	67.15
**R2W10**	0.35	0.56	1.41	9.99	1.45	1.87	13.34	71.03
**R2W11**	0.31	0.28	0.02	6.80	1.98	4.01	19.84	66.76
**R2W14**	0.29	0.46	0.49	10.75	1.11	1.58	12.46	72.86
**R3W1**	0.03	8.39	0.00	47.42	10.29	6.12	9.14	18.61
**R4WN5**	0.53	0.12	0.00	22.80	6.45	8.51	15.55	46.04
**R4WS4**	0.54	0.18	0.00	21.62	6.03	8.39	15.70	47.54
**R4WS59**	0.68	0.16	0.00	22.84	6.28	7.83	14.31	47.90
**S5**	0.26	1.85	0.00	31.10	7.11	6.84	15.52	37.31
**B7**	0.09	3.72	5.29	46.01	5.36	4.18	10.55	24.80
**B59**	0.12	2.58	0.00	34.18	6.71	6.32	13.41	36.68
**B72**	0.20	0.90	8.20	49.00	9.52	5.70	12.00	14.48
**B97**	0.12	2.77	0.00	32.04	6.43	6.05	12.66	39.93
**F03**	0.45	1.64	0.00	45.85	7.15	6.74	6.63	31.54
**F05**	0.45	2.07	0.38	26.58	7.89	6.73	9.83	46.07
**F12**	0.32	2.80	1.49	45.29	9.11	5.50	7.19	28.30
**J01**	0.50	11.37	3.22	27.35	9.36	6.47	6.12	35.62
**S01**	0.17	2.09	0.00	34.83	7.57	6.04	7.62	41.68
**S02**	0.16	1.66	0.00	32.59	7.82	7.71	15.48	34.58
**S14**	0.91	2.34	0.08	16.84	4.99	5.14	6.60	63.11
**Tahiti_GOM**	0.12	0.07	0.00	37.52	5.40	5.32	5.98	45.59
**U01**	0.10	0.90	0.00	12.61	4.60	5.10	5.10	71.59
**U08**	0.40	1.09	0.67	19.07	4.29	4.74	10.32	59.42

Since the critical parameters and acentric factor are defined for pure components, Table S10 shows these parameters for only the pseudo components (Heavy gas and SLF). Recall that these are based on the calculation of Aromatic Ring Index and then the estimation of the parameters using the developed correlations.

Table S10. Critical Properties of Heavy Gas and Single Liquid Fraction for the 35 studied crudes.

**Name**	**MW (g/mol)**	**Tc (K)**	**Pc (Bar)**	**Vc (cm3/mol)**	**Acentric factor (-)**
**B16**					
HEAVY GAS	74.35	475.92	34.70	314.45	0.241
SLF	193.00	727.20	19.04	762.37	0.600
**C02**					
HEAVY GAS	69.54	464.58	36.70	314.45	0.226
SLF	290.30	843.47	14.70	762.37	0.853
F01					
HEAVY GAS	64.60	452.49	39.04	314.45	0.210
SLF	217.56	760.31	17.50	762.37	0.670
**F07**					
HEAVY GAS	64.17	451.40	39.26	270.95	0.209
SLF	175.32	700.82	20.37	691.48	0.549
**F08**					
HEAVY GAS	64.23	451.56	39.23	271.22	0.209
SLF	178.37	698.50	19.65	710.50	0.559
**F09**					
HEAVY GAS	74.35	475.92	34.70	314.45	0.241
SLF	193.00	727.20	19.04	762.37	0.600
**F10**					
HEAVY GAS	64.36	451.88	39.16	271.78	0.209
SLF	183.45	707.80	19.37	729.25	0.574
**M1**					
HEAVY GAS	67.30	459.16	37.72	284.36	0.219
SLF	243.00	804.80	17.05	959.58	0.732
**R2W1**					
HEAVY GAS	70.44	466.75	36.30	297.79	0.229
SLF	231.00	777.02	16.79	920.41	0.707
**R2W2**					
HEAVY GAS	70.08	465.88	36.46	296.22	0.228
SLF	224.00	769.92	17.24	889.23	0.687
**R2W3**					
HEAVY GAS	71.31	468.82	35.93	301.51	0.231
SLF	233.00	780.22	16.74	928.21	0.712
**R2W10**					
HEAVY GAS	73.75	474.54	34.94	311.90	0.239
SLF	258.00	806.85	15.53	1039.45	0.779
**R2W11**					
HEAVY GAS	72.11	470.70	35.60	304.90	0.234
SLF	237.00	782.44	16.39	947.83	0.724

**R2W14**					
HEAVY GAS	73.29	473.45	35.12	309.92	0.238
SLF	261.00	802.76	14.97	1059.37	0.792
**R3W1**					
HEAVY GAS	68.81	462.83	37.02	290.81	0.223
SLF	180.00	712.42	20.30	705.84	0.562
**R4WN5**					
HEAVY GAS	69.97	465.62	36.51	295.77	0.227
SLF	242.00	790.01	16.27	967.83	0.736
**R4WN118S**					
HEAVY GAS	68.34	461.70	37.24	288.81	0.222
SLF	238.00	789.47	16.70	946.59	0.724
**R4WS4**					
HEAVY GAS	69.20	463.78	36.85	292.48	0.225
SLF	236.00	784.62	16.64	940.31	0.720
**R4WS26**					
HEAVY GAS	71.26	468.70	35.95	301.29	0.231
SLF	257.00	804.82	15.51	1035.81	0.777
**R4WS59**					
HEAVY GAS	69.36	464.16	36.78	293.17	0.225
SLF	226.00	777.10	17.43	893.17	0.691
**S5**					
HEAVY GAS	74.35	475.92	34.70	314.45	0.241
SLF	193.00	727.20	19.04	762.37	0.600
**B07**					
HEAVY GAS	75.26	478.02	34.35	318.33	0.244
SLF	212.90	754.43	17.78	844.01	0.657
**B59**					
HEAVY GAS	72.33	471.21	35.51	305.84	0.235
SLF	208.00	742.50	17.73	829.12	0.645
**B72**					
HEAVY GAS	76.29	480.38	33.97	322.72	0.247
SLF	189.00	714.32	18.86	753.25	0.591
**B97**					
HEAVY GAS	72.80	472.32	35.32	307.84	0.236
SLF	201.90	740.69	18.52	797.63	0.625
**F03**					
HEAVY GAS	63.79	450.46	39.46	269.35	0.207
SLF	188.30	719.57	19.32	744.19	0.587
**F05**					
HEAVY GAS	64.46	452.14	39.11	272.22	0.210
SLF	218.30	771.69	18.11	856.59	0.668
**F12**					

HEAVY GAS	64.09	451.21	39.30	270.63	0.208
SLF	194.77	730.80	18.99	768.52	0.605
**J01**					
HEAVY GAS	69.94	465.55	36.52	295.65	0.227
SLF	229.00	780.69	17.26	906.02	0.699
**S01**					
HEAVY GAS	67.27	459.09	37.73	284.23	0.219
SLF	191.00	718.52	18.80	760.02	0.596
**S02**					
HEAVY GAS	72.16	470.82	35.58	305.11	0.234
SLF	193.50	720.74	18.54	771.52	0.604
**S14**					
HEAVY GAS	66.29	456.68	38.20	280.03	0.215
SLF	181.00	712.74	20.14	710.99	0.565
**Tahiti_GOM**					
HEAVY GAS	67.85	460.50	37.46	286.71	0.220
SLF	237.10	797.21	17.28	934.47	0.717
**U01**					
HEAVY GAS	65.42	454.53	38.63	276.32	0.213
SLF	212.40	767.80	18.70	828.37	0.651
**U08**					
HEAVY GAS	71.47	469.19	35.87	302.17	0.232
SLF	195.50	723.49	18.40	779.74	0.609

For the Peng-Robinson Equation of State to model these crude oils, the binary interaction parameters (k_{ij}) are needed. In this case, the binary interaction parameters are adjusted to fit the density and phase behavior of B59 and then used for the rest of crude oils. Please note, that further adjustment can improve the individual performance for the different phase behaviors. The used k_{ij} are summarized in Table S11.

Table S11. Non-zero binary interaction parameters used for the 35 crudes.

k_{ij}	**N_2**	**CO_2**	**H_2S**	**C1**	**C2**	**C3**	**C4+**
**N_2**	-						
**CO_2**	0.000	-					
**H_2S**	0.100	0.100	-				
**C1**	0.020	0.120	0.100	-			
**C2**	0.060	0.150	0.100	-0.017	-		
**C3**	0.060	0.150	0.100	0.003	0.000	-	
**C4+**	0.100	0.150	0.080	0.000	0.000	0.000	-
**SLF**	0.210	0.140	0.050	0.040	0.000	0.000	0.000

The list of components studied and analyzed for the development of critical properties correlations are summarized in Table S12 below. ARI can be calculated from the molecular weight and density.

Table S12. List of components studied and are plotted in the parity plots.

Component	$\boldsymbol{\rho}$ at 20°C, (g/cm³)	MW	Component	$\boldsymbol{\rho}$ at 20°C, (g/cm³)	MW
n-butane	0.5791	58.12	Propylcyclohexane	0.7940	126.24
n-pentane	0.6260	72.15	Butylcyclohexane	0.7993	140.27
n-hexane	0.6605	86.18	Benzene	0.8780	78.11
n-heptane	0.6857	100.21	Toluene	0.8685	92.14
n-octane	0.7031	114.23	Ethylbenzene	0.8678	106.17
n-nonane	0.7180	128.26	Propylbenzene	0.8630	120.20
n-decane	0.7302	142.29	Butylbenzene	0.8610	134.22
n-undecane	0.7400	156.31	Pentylbenzene	0.8583	148.24
n-dodecane	0.7585	170.34	Hexylbenzene	0.8581	162.27
n-tridecane	0.7571	184.37	Heptylbenzene	0.8576	176.29
n-tetradecane	0.7627	198.39	Octylbenzene	0.8562	190.32
n-pentadecane	0.7680	212.42	Nonylbenzene	0.8557	204.35
n-hexadecane	0.7729	226.45	Decylbenzene	0.8551	218.38
n-heptadecane	0.7765	240.48	Undecylbenzene	0.8548	232.40
n-octadecane	0.7805	254.50	Dodecylbenzene	0.8556	246.43
n-nonadecane	0.7844	268.53	Tridecylbenzene	0.8545	260.46
n-eicosane	0.7871	282.56	Tetradecylbenzene	0.8553	274.48
n-henicosane	0.7906	296.58	Pentadecylbenzene	0.8540	288.51
n-docosane	0.7981	310.61	Hexadecylbenzene	0.8541	302.54
Cyclopentane	0.7456	70.14	o-xylene	0.8799	106.16
Methylcyclopentane	0.7491	84.16	m-xylene	0.8643	106.16
Ethylcyclopentane	0.7667	98.19	Pentene	0.6402	70.13
Propylcyclopentane	0.7768	112.21	Hexene	0.6740	84.16
Butylcyclopentane	0.7851	126.24	Heptene	0.6971	98.19
Pentylcyclopentane	0.7954	140.27	Octene	0.7140	112.24
Hexylcyclopentane	0.8006	154.29	Pyridine	0.9819	79.10
Heptylcyclopentane	0.8051	168.32	Aniline	1.0200	93.13
Octylcyclopentane	0.8088	182.35	Indole	1.1700	117.15
Nonylcyclopentane	0.8121	196.38	Quinoline	1.0900	129.16
Decylcyclopentane	0.8149	210.40	Azetidine	0.8470	57.09
Undecylcyclopentane	0.8145	224.43	Pyrrole	0.9670	67.09
Dodecylcyclopentane	0.8197	238.46	Diphenyl sulfide	1.1130	186.27
Tridecylcyclopentane	0.8217	252.48	Dibenzothiophene	1.2520	184.26
Tetradecylcyclopentane	0.8235	266.51	3-Methylbenzothiophene	1.1060	198.29
Pentadecylcyclopentane	0.8252	280.54	Naphthalene	1.1400	128.17
Hexadecylcyclopentane	0.8267	294.56	Anthracene	1.2500	178.23
Ethylcyclohexane	0.7884	112.21	Phenanthracene	1.1800	178.23
